# Supplementary material for: Theta Rhythmic Neuronal Activity and Reaction Times Arising from Cortical Receptive Field Interactions during Distributed Attention
Source: Curr Biol. 2018 Aug 6;28(15):2377–2387.e5. doi: 10.1016/j.cub.2018.05.086 (PMC6089835; doi:10.1016/j.cub.2018.05.086)
Supplement: Document S1. Figures S1–S5 [file mmc1.pdf]

Current Biology, Volume *28*

Supplemental Information

Theta Rhythmic Neuronal Activity and Reaction Times Arising from Cortical Receptive Field Interactions during Distributed Attention

Ricardo Kienitz, Joscha T. Schmiedt, Katharine A. Shapcott, Kleopatra Kouroupaki, Richard C. Saunders, and Michael Christoph Schmid

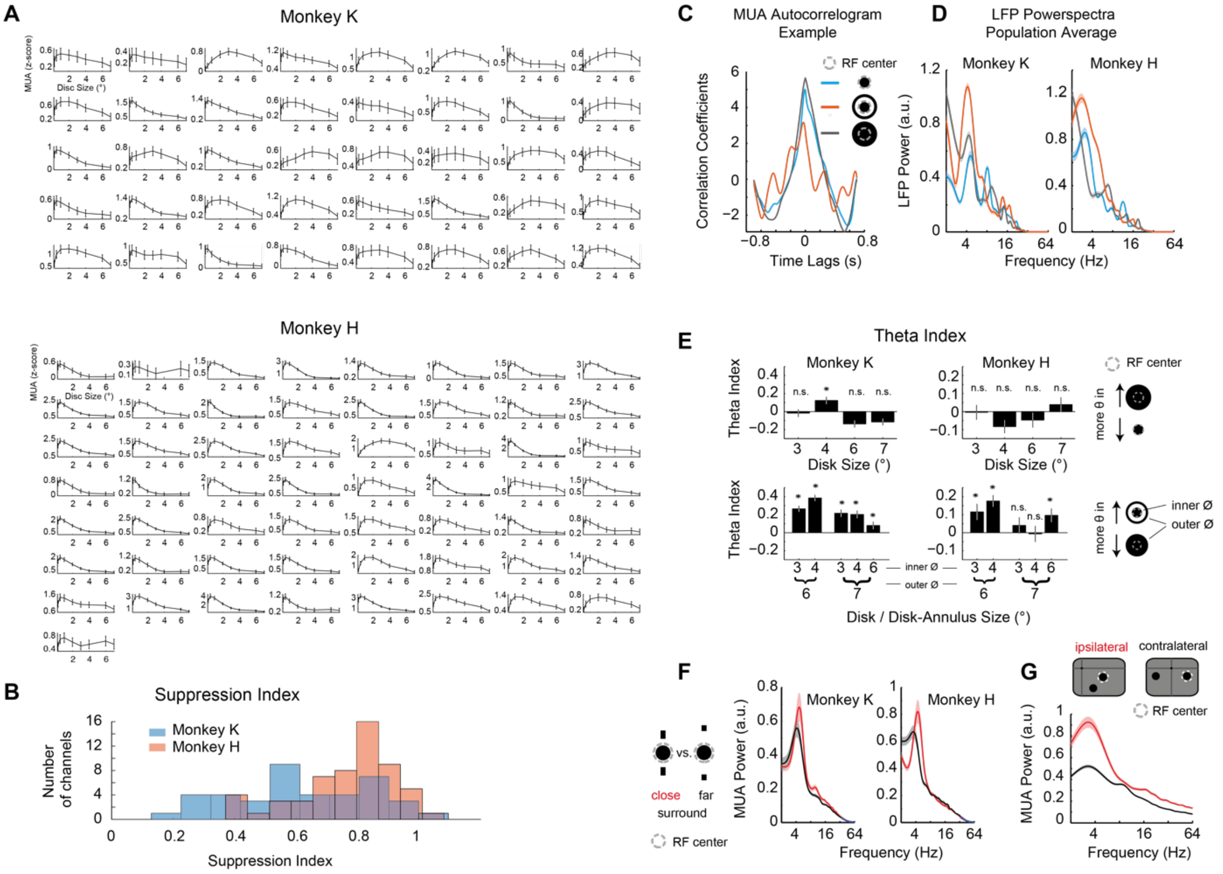

Figure S1. Surround suppression and theta oscillations, Related to Figure 1.

(A) Size tuning curves of all included channels (average across trials) from monkey K (top panel) and monkey H (lower panel).

(B) Distribution of SI values from monkey K (blue, mean = 0.62 ± 0.03, n = 40) and monkey H (red, mean = 0.77 ± 0.02, n = 57).

(C) Example MUA autocorrelogram from the same channel as in **Figure 1B-C** with the same color conventions (small 2° disk (blue); large 6° disk (gray); 2° disk with 4-6° annulus (orange), see also little insets). Note the oscillation in the disk-annulus condition only (orange) that resembles the findings of the spectral analysis in **Figure 1B.**

(D) Population average of LFP powerspectra across channels for monkey K (left panel) and H (right panel), same color convention as in (C) and in **Figure 1B-C**. Note the presence of a peak in the theta range and that strongest theta power is found in the disk-annulus condition (orange).

(E) Upper Row: Distributions of theta indices comparing the theta power of a 2° disk (diameter), roughly corresponding to the RF center, vs. lager disks also stimulating the RF surround (3°, 4°, 6°, 7° in diameter). Positive values indicate higher theta power for the larger disk (presence of inhibition). Note that no theta peak was visible in the powerspectra for both small and large disks (**Figure 1**) indicating that higher power values represent unspecific over-all power shifts.

Lower Row: Distributions of theta indices comparing the theta power of large disks and disk-annulus stimuli with the same outer diameter, i.e. same spatial extent into

suppressive surround. Positive values indicate higher theta power for the disk-annulus stimuli.

(F) Powerspectra show MUA power averaged across channels during the passive viewing disk-flanker task (see **Figure S2**) comparing conditions where the bars falling in the suppressive surround are close to the central disk (red lines, 1° gap, 1° bar length) and further away (black lines, 2° gap, 0.5° bar length) from monkey K (left) and H (right), showing lower theta power when suppression is reduced.

(G) Powerspectrum showing MUA power averaged across channels from monkey K recorded during a passive viewing task (1s fixation, 1s stimulus presentation) comparing two conditions where two disks (black, 2° diameter) are either displayed in one hemifield (red lines, *ipsilateral*) vs. both hemifields (black lines, *contralateral*).

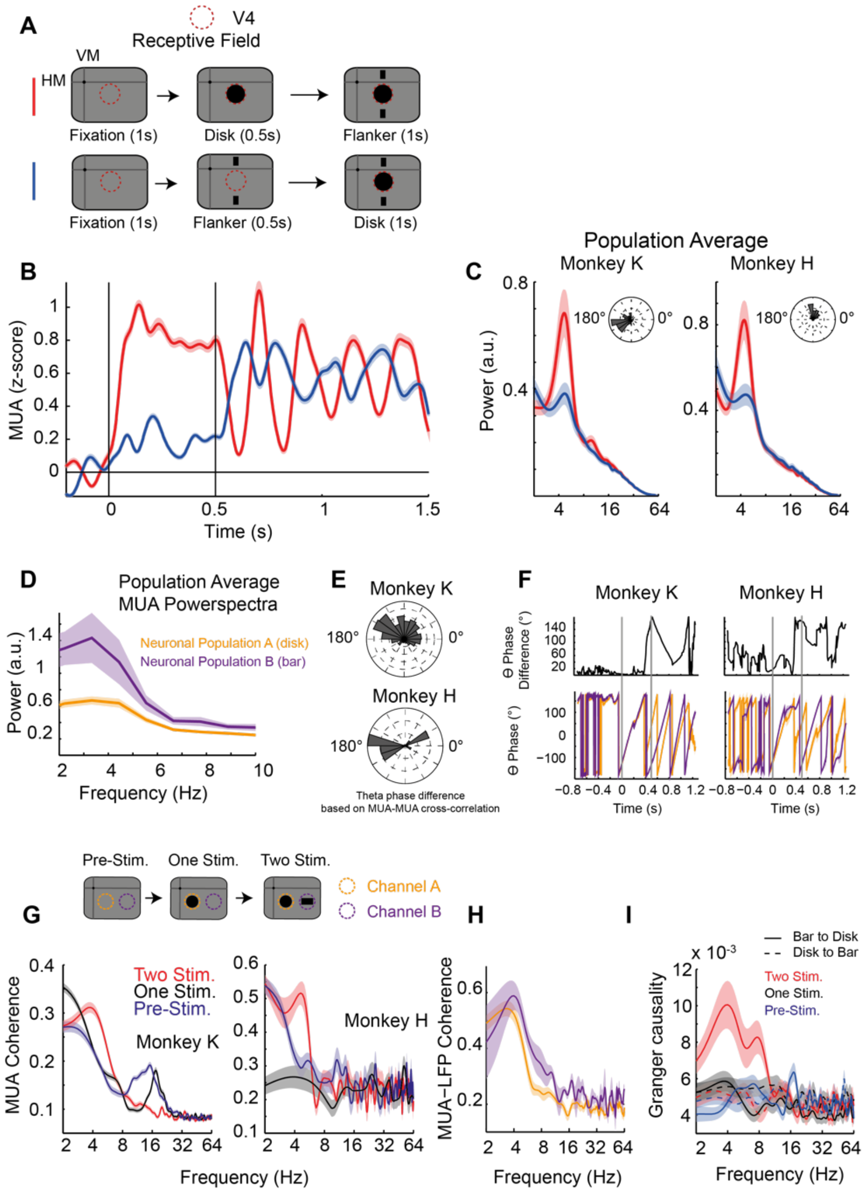

Figure S2. Stimulus timing and location controls phase, Related to Figure 2.

(A) Experimental design involving passive viewing disk-flanker stimulation providing the data shown in **B-C**. After 1s of fixation either the disk (in RF center, red) or the flanker (in RF suppressive surround, blue) appeared first, followed by the respective other stimulus.

(B) MUA responses from one example channel of monkey K to the two different stimulation conditions (averaged across trials), same color conventions as in (A).

(C) Population powerspectra of monkey K (left panel) and monkey H (right panel) with phase plots showing the channel-wise theta phase-differences between the two conditions of monkey K (left panel) and monkey H (right panel) across channels (mean: -168.6° ± 6.7°, n = 40 for monkey K; mean: 93.8° ± 7.3°, n = 57), same color conventions as in (A).

(D) Population average powerspectra across MUA channels of monkey K of the task involving the sequential presentation of disk and bar as displayed in **Figure 2**.

(E) Rose plots showing the distribution of phase differences between the two adjacent

neuronal populations as displayed in **Figure 2** from monkey K (left) and H (right) based on cross-correlation lag-analysis (see Methods).

(F) Lower panels depict phase plots showing the MUA theta phase across time recorded during the disk-bar task (**Figure 2**) from monkey K (left panel) and monkey H (right panel). Upper panels depict the corresponding phase difference between both neuronal groups (0°: in phase, 180°: out-of-phase). Vertical lines highlight stimulus onsets. Same color conventions as in Figure 2 and S2D.

(G) Spectra depict MUA-MUA coherence between the two neighboring neuronal populations as displayed in **Figure 2** from monkey K (left) and H (right) averaged across MUA channel combinations for the prestimulus fixation period (blue lines), the one-stimulus only period (black lines) and the two-stimulus period (red lines). Note that theta coherence is only present when the neighboring stimuli are displayed (red liens).

(H) Spectrum depicts the MUA-LFP coherence from monkey K during the two-stimulus period of the disk-bar task (**Figure 2, S2G**). Same color conventions as in **Figure 2, S2D.**

(I) Granger causality spectrum from monkey K for the same conditions as in (**G**). Solid lines depict granger causal influences from the bar-selective to the disk-selective MUA channels (dashed line vice versa). Note that granger causal interactions are present only during the two-stimulus period and are directed from bar- to disk-selective MUA channels.

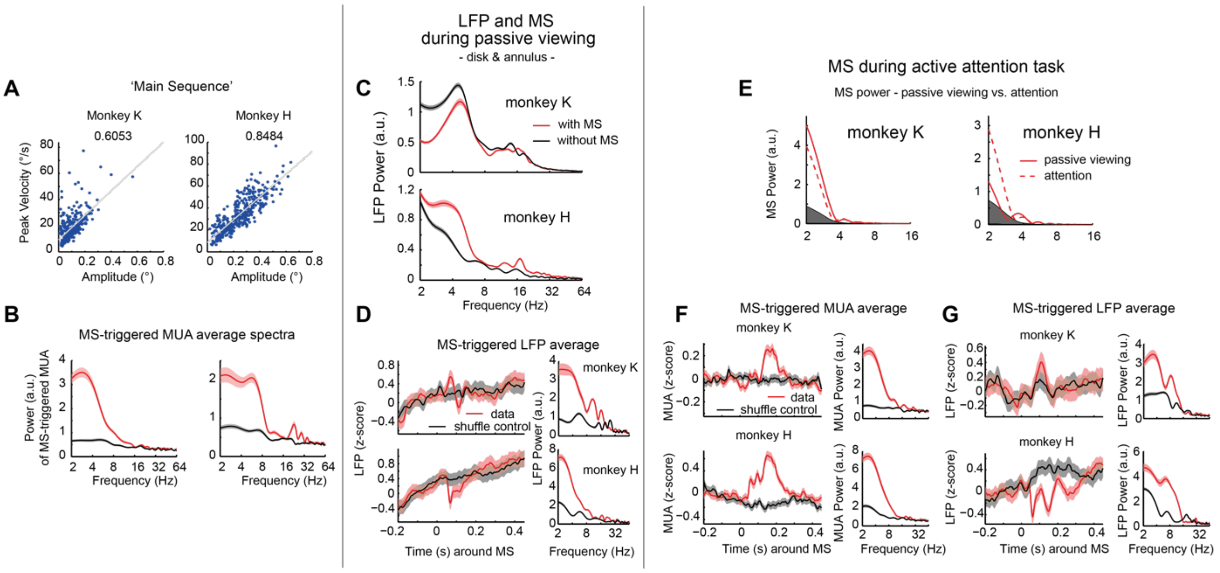

Figure S3. Microsaccade (MS) main sequence and MS analysis, Related to Figure 3.

(A) Main sequence of microsaccades (MS) for monkey K (left) and H (right) showing the relation of amplitude and peak velocity of each MS and the respective correlation values across MS.

(B) Average Powerspectra of MS-triggered MUA computed during the same task as in **Figures 1, 3** after MS onset for monkey K (left) and H (right) showing the actual data (red) and trial-shuffled control data (gray).

(C) Average LFP powerspectra from monkey K (upper panel) and H (lower panel) based on trials with (red lines) and without MS (black lines) recorded during the disk-annulus passive viewing task (as also displayed in **Figures 1, 3**).

(D) Left panels show MS-triggered LFP average (actual data: red lines, shuffle control: black lines), right panels depict powerspectra of the MS-triggered LFP average. Upper rows show data from monkey K, lower panels from monkey H.

(E) Powerspectra of MS during the disk-annulus passive viewing task (solid red lines) and the attention task (dashed red lines) from monkey K (left) and H (right). Shaded area depicts 95% confidence interval based on shuffle controls.

(F) Same as (D), but for MS-triggered MUA average recorded during the attention task.

(G) Same as (D), (F), but for MS-triggered LFP average recorded during the attention task.

Shaded areas depict SEM, if not stated otherwise.

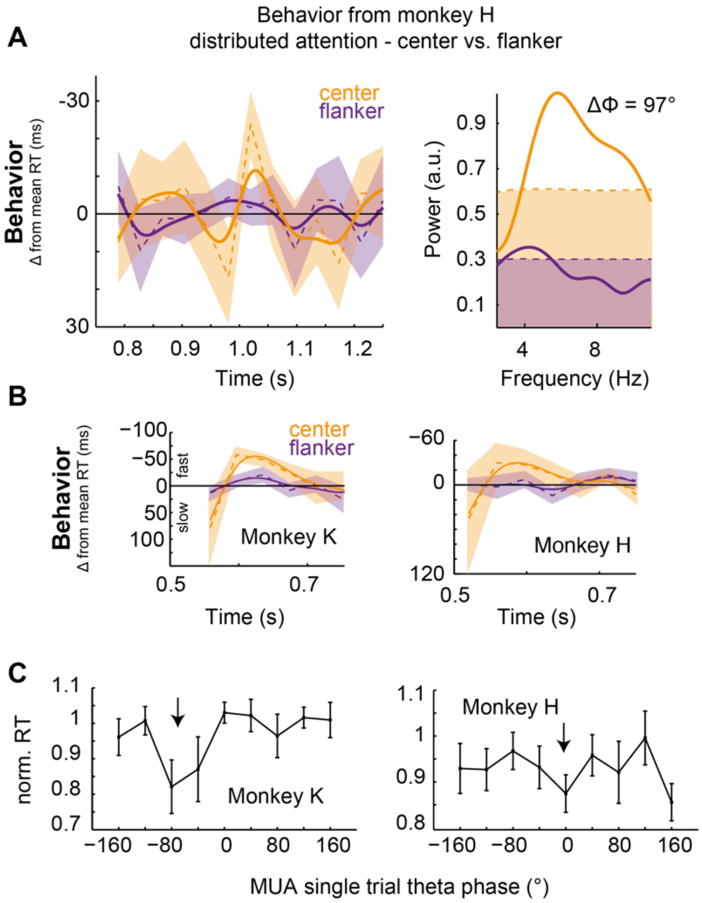

Figure S4. Reaction time data of Monkey H, Related to Figure 4.

(A) Left panel showing the reaction time (as deviation from mean) as a function of target delay for the distributed attention condition in response to the center (solid line) and flanker target position (dashed line). Non-smoothed RT data shown as thin dashed lines, shadings around lines depict SEM. Right panel depicts powerspectra of the respective non-smoothed RT data and their phase difference (97°). Shadings represent 95% confidence intervals based on shuffled surrogate data.

(B) Left panel shows RT data including the part excluded elsewhere due to a masking effect from monkey K (first data point not discernible due to 0% performance). Right panel shows same data from monkey H. Same color convention as in (A).

(C) Average normalized reaction times are plotted against MUA theta phase (0° corresponding to the oscillation's peak) on a single trial level estimated at target presentation time from monkey K (left) and H (right). Error bars indicate SEM, arrows indicate MUA theta phase of fast RT.

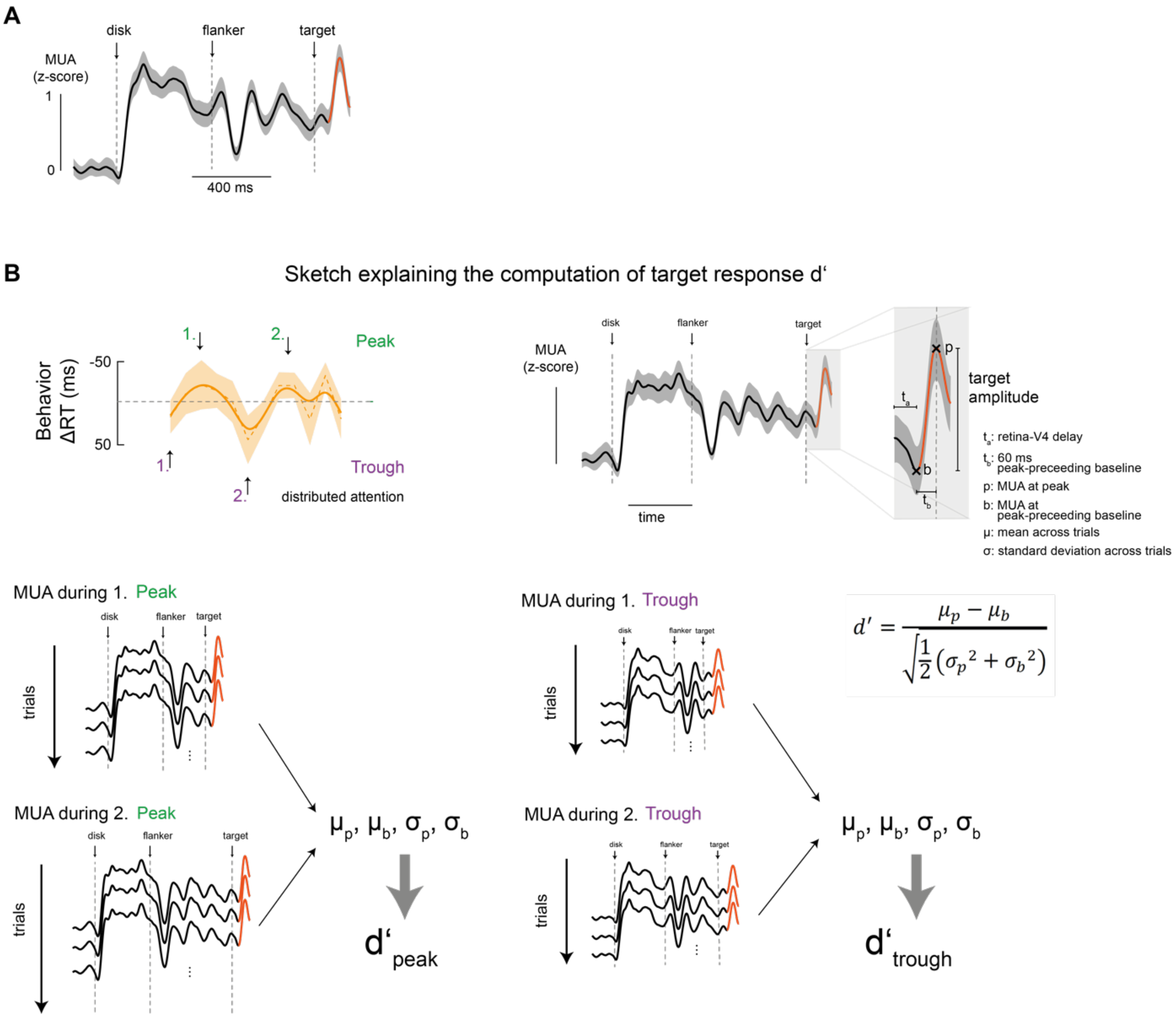

$$d' = \frac{\mu_p - \mu_b}{\sqrt{\frac{1}{2}(\sigma_p{}^2 + \sigma_b{}^2)}}$$

Figure S5. MUA target response and the computation of d', Related to Figure 6.

(A) Example MUA channel, averaged across trials, recorded during the distributed attention task where the target presentation falls onto a trough / rising part of the ongoing oscillation. Compare to **Figure 6A**.

(B) Sketch illustrating the computation of the d' quantifying the target response. Upper left panel shows the RT trace of monkey K highlighting the peaks and troughs and the corresponding SOA conditions that were used to analyze MUA and its target responses during the distributed attention task. Upper right panel explains how the quantification of target responses and d' was calculated. The peak amplitude was defined as the amplitude difference between the peak and the baseline that preceded the peak by 60 ms. It was then related to its variation across trials as shown in the d' equation. The lower panels illustrate that the target responses were quantified across trials for the selected RT peak and trough SOAs.
